# Airborne Intelligent System for Abnormal Pig Behavior Identification and Locking

**DOI:** 10.3390/ani16101506

**Published:** 2026-05-14

**Authors:** Yun Wang, Haopu Li, Zhihui Xiong, Yuanmeng Hu, Guangying Hu, Zhenyu Liu

**Affiliations:** 1 College of Agricultural Engineering, Shanxi Agricultural University, Jinzhong 030801, China; 17836205306@163.com (Y.W.); lihp0307@163.com (H.L.); 18434802788@163.com (Y.H.); 2 College of Information Science and Engineering, Shanxi Agricultural University, Jinzhong 030801, China; 18434183406@163.com; 3 College of Zoological Sciences, Shanxi Agricultural University, Jinzhong 030801, China; huguangying1968@163.com; 4 Dryland Farm Machinery Key Technology and Equipment Key Laboratory of Shanxi Province, Jinzhong 030801, China

**Keywords:** aerial platform, embedded system, target locking, deep learning, identity recognition

## Abstract

Intensive pig farming presents substantial challenges for individual health monitoring due to high stocking densities, complex occlusion scenarios, and the need for continuous real-time surveillance. Existing monitoring approaches rely heavily on manual inspection, which is labor-intensive and prone to delayed detection of abnormal behaviors and disease symptoms. This study proposes an embedded intelligent monitoring system integrating a pan-tilt gimbal platform with an improved multi-object tracking and anomaly detection framework for automated pig health surveillance. The system employs a modified Periodfill_DeepSORT algorithm that incorporates a ReID network with appearance features and motion prediction trajectories to maintain identity consistency under occlusion and re-entry scenarios. For anomaly detection, a lightweight YOLOv8-based network was trained on 772 abnormal samples across three behavioral categories: movement abnormalities, postural abnormalities, and disease-related abnormalities. Experimental results demonstrate that the Periodfill_DeepSORT algorithm achieves a Multiple Object Tracking Accuracy (MOTA) of 95.34%, a Multiple Object Tracking Precision (MOTP) of 94.77%, and an IDF1 score of 96.88%, with only 12 identity switches across 2000 frames involving 12 targets—27 fewer than the standard DeepSORT algorithm. In occlusion scenarios, MOTA improved from 61.1% to 78.3%. The anomaly detection network achieves an overall detection accuracy of 94.5%, representing an 8.8 percentage point improvement over the baseline model, with recognition accuracies of 96.2% for movement abnormalities, 94.1% for postural abnormalities, and 92.8% for disease-related abnormalities. The system operates at 90 frames per second on embedded hardware with a power consumption of 3.2 watts and a startup time of approximately 1 s, with gimbal angle errors maintained within 3°. These results demonstrate the system’s effectiveness and practical feasibility for real-time intelligent health monitoring in intensive livestock farming environments.

## Simple Summary

Intensive pig farming faces significant challenges in animal health monitoring due to high stocking densities and complex environments. Traditional manual observation methods are inefficient and often fail to detect early signs of illness or abnormal behavior in time. This study presents an intelligent monitoring system that combines an embedded pan-tilt camera platform with artificial intelligence to automatically track individual pigs and detect abnormal behaviors in real time. The system uses an improved tracking algorithm that maintains continuous identity locking of individual pigs even when they are temporarily hidden behind other animals or objects. In tests involving 12 pigs over 2000 video frames, the system successfully identified all individuals and maintained stable tracking throughout. The system experienced only 12 instances of identity confusion, far fewer than existing methods. For health monitoring, the artificial intelligence model was trained to recognize three types of abnormal behaviors: unusual movement, abnormal posture, and disease-related signs. The model achieved an overall detection accuracy of 94.5%, with movement abnormalities detected at 96.2% accuracy. The system operates at 90 frames per second on low-power embedded hardware consuming only 3.2 watts, making it suitable for practical deployment in commercial pig farms to support early disease warning and improve animal welfare.

## 1. Introduction

The automatic recognition and monitoring of abnormal behaviors in pigs face numerous challenges in practical applications within resource-saving pig farming as the scale of swine production continues to expand. Pig management using physical observation is impractical because of the scale of modern pig production systems [1]. Abnormal behaviors in pigs are often important indicators of early disease symptoms, and timely identification of these behavioral patterns is of significant value for preventing disease spread and reducing economic losses. It is increasingly important to employ a continuous and effective monitoring system for detecting and tracking individual animals in large-scale farms [2]. Precision livestock farming aims to individually and automatically monitor animal activity to ensure their health, well-being, and productivity [3]. Abnormal behavior recognition not only helps detect potential health issues early but also significantly improves livestock safety levels in farming environments. These abnormal behaviors include irregular activity levels, abnormal lying postures, and unusual clustering, which are often early warning signals of disease outbreaks or stress responses. Further application of the optimal model could facilitate the detection of abnormal behaviors such as smothering and piling [4]. Unidentified abnormal behaviors can lead to rapid disease spread within the group, and reduce potential economic losses [5]. However, accurately identifying pig behavior in complex pigsty environments presents many challenges. In complex pigsty environments, due to pigs’ stress reactions and frequent obstructions, it is challenging to count them accurately and automatically [6]. Moreover, the complex environment of pig farms and the dynamic nature of pig behaviors can lead to issues such as false positives, false negatives, and identity switches, which pose significant challenges for the monitoring of individual pig behaviors [7]. Additionally, errors in pig identification and tracking can propagate to later frames [8]. These factors make it difficult for traditional single detection or tracking methods to achieve stable results.

Traditional pigsty monitoring systems can be roughly divided into three categories. The first type is fixed camera monitoring. This involves using statically installed cameras to monitor specific areas. The main technical drawback is the inherent limitation of spatial coverage, where the fixed field of view cannot adapt to the dynamic activity trajectories of pigs, leading to extensive monitoring blind spots. This is important as the image dimensions can vary with different positioning of broilers below the camera [9]. When a target pig moves out of the preset monitoring range, the system immediately loses tracking ability. The second type is fixed swivel cameras. In this setup, the camera is fixed but includes a rotating function, which expands the monitoring range to some extent. However, there are significant challenges when processing back-facing target recognition, as when a pig faces away from the camera, facial feature information is completely absent, and re-identification algorithms cannot obtain valid identity verification data. The third type is UAV-mounted fixed cameras. This uses drone platforms equipped with fixed cameras to achieve mobile monitoring. Although this model overcomes the limitation of fixed installation positions, it has significant mobility defects. The drone must frequently adjust its flight posture to keep the target in the center of its field of view, which results in a sharp increase in energy consumption and a substantial rise in operational difficulty. But it might not be suitable for applications like cattle counting, where the target of interest is constantly moving [10]. In narrow corridors or complex obstacle environments, the drone’s maneuverability is limited, making it difficult to effectively lock onto the target. Frequent flight path adjustments can easily disrupt the farming environment, affecting the normal behavior of the pigs. Traditional methods struggle to achieve continuous tracking and individualized monitoring of a single abnormal pig, limiting the practicality of abnormal behavior recognition. The motion cues are first used in linear assignment to reduce the interference of occlusion and deformation issues, and when the motion model fails to track, the appearance cues can recover the identity to deal with long occlusion [11]. This makes making robust and adaptable tracking methods crucial for maintaining reliable performance in diverse and dynamic conditions [12].

Despite these advances, several critical gaps remain unaddressed in current livestock tracking systems:

1. Existing methods struggle with severe occlusions in high-density farming environments, where animals frequently overlap and obscure each other.
2. Most tracking algorithms fail to maintain identity consistency during prolonged occlusions, leading to frequent ID switches.
3. Current approaches lack efficient conflict resolution mechanisms when multiple tracking hypotheses compete for the same detection.
4. The computational complexity of state-of-the-art trackers limits their deployment on resource-constrained edge devices commonly used in precision livestock farming.

These limitations motivate the development of a robust, efficient tracking solution specifically designed for the challenging conditions of intensive livestock monitoring.

Computer vision-based tracking systems offer a promising alternative by enabling continuous, non-invasive monitoring of animal activities. Recent advances in deep learning have significantly improved multi-object tracking (MOT) accuracy. DeepSORT combines appearance features with Kalman filter-based motion prediction, achieving a balance between accuracy and efficiency. Recent state-of-the-art methods have further pushed the boundaries: ByteTrack improves recall by associating low-confidence detections; OC-SORT enhances motion modeling through observation-centric strategies; StrongSORT integrates multiple appearance models for robust matching. However, these methods face challenges in livestock monitoring: ByteTrack’s reliance on detection confidence is problematic when animals exhibit similar appearances; OC-SORT struggles with prolonged occlusions common in high-density farming; StrongSORT’s computational complexity limits edge deployment for UAV-based monitoring.

To address the aforementioned technical challenges, this study proposes three key innovations to construct an efficient, low-power aerial intelligent locking system: (1) Lightweight system integration on resource-constrained embedded platforms. This study proposes a lightweight target recognition and locking system tailored for constrained computing environments. Through architectural optimization, efficient deployment is achieved on a microcontroller combined with a dedicated AI accelerator. System innovations include: introducing a new module called Squeeze aggregated excitation (SaE) [13] to enhance the network’s representational capability, improving model performance with only a slight increase in parameters and enabling deployment on lightweight devices; improving the ReID feature network by adding an Adaptive (ADA) module, allowing it to adapt to targets of various sizes and expanding the model’s data adaptability; and constructing a Raspberry Pi + AI accelerator collaborative architecture to achieve the organic separation of intelligent decision-making and precise execution. (2) Periodfill_DeepSORT dual-locking mechanism and conflict coverage strategy. With the rapid development of object tracking technology [14], this study proposes an integrated method that develops the Periodfill_DeepSORT target-locking algorithm to address identity switching and trajectory interruption issues in complex farming environments. The algorithm’s core innovations include: a dual-locking mechanism—the primary locker (DeepSORT) handles long-term trajectory association and identity recognition, while the auxiliary locker (KCF) focuses on short-term precise localization, providing continuous positional data when the primary locker fails; a conflict coverage mechanism—by monitoring all active lockers’ target regions in real time, the system performs conflict analysis upon detecting overlapping locks, ensuring that the same target is not recognized repeatedly; and a trajectory compensation strategy—using a lightweight deep learning model for visual object detection, the improved locking algorithm compensates for temporary positional continuity when detection fails, ensuring target ID consistency. To enhance the adaptability of tracking technology for group-housed pigs and to reduce pig identity switching [15], the proposed locking algorithm is improved based on the concept of the track fusion module [16], enabling the system to maintain trajectory association and target stability even during detection failures or reconnections. (3) Collaborative control architecture of aerial hovering and active gimbal locking. This research innovatively introduces a technological pathway in which an aerial vehicle is equipped with an intelligent gimbal system, achieving functional decoupling and coordinated optimization between the aerial platform and the tracking device. The core technical advantages of this architecture include the following: The aerial vehicle only needs to maintain a relatively stable hovering state, greatly reducing flight operation complexity and energy consumption requirements; the onboard intelligent gimbal system can independently perform target-locking operations, allowing the camera to achieve 360° horizontal rotation and 180° vertical tilt, ensuring that the target remains within the optimal field of view at all times; and with real-time decision-making enabled by the embedded AI accelerator, the gimbal can predict the target’s motion trajectory and actively adjust its posture, achieving truly intelligent continuous locking.

The main contributions of this paper are summarized as follows:

1. We propose an enhanced tracking framework that achieves a 5.4% improvement in MOTA and a 69.2% reduction in ID switches compared to baseline DeepSORT.
2. We introduce a conflict coverage mechanism that reduces false associations by 67.8% in heavily occluded scenarios.
3. We develop a lightweight system achieving real-time performance (90 FPS) on edge devices, enabling practical farm deployment.
4. We conduct extensive experiments demonstrating statistically significant improvements multiple over six state-of-the-art trackers.

This study simultaneously addresses the reduction in identity switches and the problem of starting new tracks [17]. The data generated by tracking is used in many other downstream tasks [18], thereby significantly improving the robustness of target detection and locking. The system markedly reduces computational load and power consumption while maintaining acceptable recognition accuracy and real-time performance, providing reliable visual perception capabilities for aerial platforms. For example, when applied to pig farm monitoring devices, and then based on digital image processing technology and machine learning technology for animal behaviour recognition [19], the mounted system enables precise localization and continuous locking, offering effective support for subsequent observation and management.

## 2. Locking System Architecture

In resource-saving pig farming monitoring scenarios, the complexity of group behaviors—characterized by frequent mutual occlusions and dense collective activities—imposes stringent requirements on the robustness of the locking algorithm. Such application scenarios require locking algorithms that can effectively handle complex situations, such as when targets suddenly appear or disappear [10]. Additionally, considering the strict size and weight limitations of equipment carried by mobile monitoring platforms like drones, and the limited hardware computing resources, we are prompted to deeply optimize the locking algorithm model to balance computational efficiency with locking performance.

**Figure 1:**
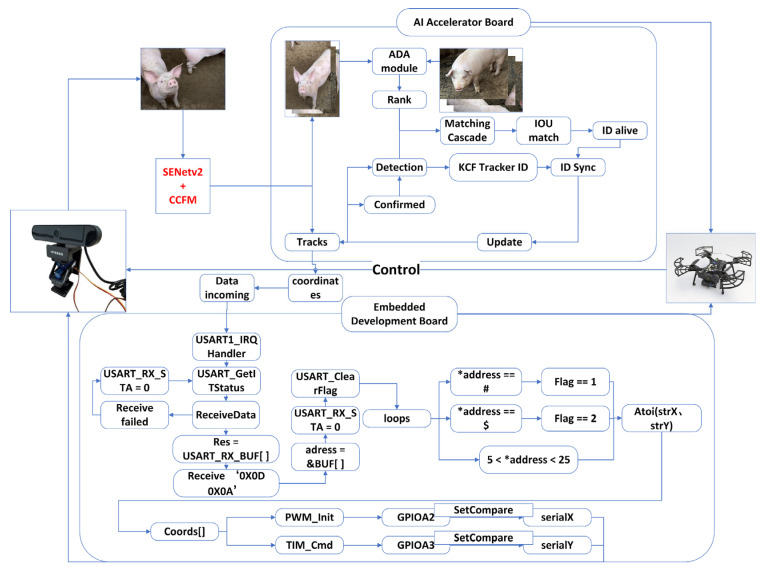
Total System Architecture. *address refers to the current character in the buffer; # and $ are delimiter markers used to distinguish the *X*- and *Y*-coordinate fields in the serial data stream. Components highlighted in red indicate the modules proposed or improved in this work.

The airborne abnormal behavior recognition and locking system developed in this study establishes a collaborative operational architecture in which a Raspberry Pi combined with an AI accelerator serves as the intelligent decision-making core, and a precision gimbal functions as the execution terminal. The system is tested with a minimum benchmark of continuous operation for 12 h. As shown in Figure 1, this architecture overcomes the traditional technological limitations of blending cognition and execution in monitoring systems, realizing the organic separation and efficient collaboration between intelligent decision-making and precise execution. The implementation part mainly includes the design and implementation of serial communication [20], and its stability is carefully analyzed [21].

The core part of the system consists of an embedded computing platform formed by the Raspberry Pi main controller and the AI accelerator Hailo-8L M.2, responsible for core functions such as visual information processing, abnormal behavior recognition, target positioning calculation, and trajectory prediction. The core control unit of the system is responsible for overall system scheduling, data transmission, and gimbal drive control. The AI acceleration board, serving as a dedicated computation accelerator, integrates an optimized Periodfill_DeepSORT locking algorithm and an improved ReID feature network based on a lightweight deep learning architecture, providing efficient abnormal behavior recognition and target locking capabilities. By collaborating with the Raspberry Pi and AI board, the system can process video stream data from the gimbal camera in real-time, accurately identify individual abnormal pigs, and deliver precise gimbal control instructions through a control pipeline. The edge part of the system consists of a precision dual-axis gimbal system, driven by the Raspberry Pi main controller, focusing on executing high-precision target locking actions according to the “brain’s” commands. The gimbal system uses the STM32F407 microcontroller as the motion control core, receiving control instructions from the Raspberry Pi and precisely driving the horizontal and pitch axis servos through PWM signals, enabling 360° horizontal rotation and 180° vertical pitching, providing full-range locking capabilities [22]. This architecture ensures the perfect combination of intelligent decision-making from the Raspberry Pi brain and precise execution from the gimbal, achieving seamless collaboration from recognition to physical locking.

### 2.1. Dual-Axis Gimbal Control Optimization and Structural Design

The dual-axis gimbal system, as the precise executor of embedded core instructions, achieves a precision locking capability with angle error control within 3° through direct drive by the controller. The gimbal’s mechanical structure uses lightweight materials, ensuring that the overall weight fully meets the drone’s payload requirements. The gimbal control system adopts a layered control architecture: the Raspberry Pi serves as the upper-level controller, responsible for receiving recognition results from the AI acceleration board, performing coordinate transformations and trajectory planning, and generating gimbal control instructions; the STM32F407VGT6 microcontroller acts as the lower-level executor, dedicated to the precise generation of PWM signals and real-time servo control. The Raspberry Pi sends target coordinate instructions to the STM32 via UART serial communication, and the STM32 immediately responds by generating precise PWM control signals to drive the horizontal and pitch axis servos for precise locking actions. This layered control architecture ensures efficient collaboration and precise response from AI recognition to gimbal execution.

**Figure 2:**
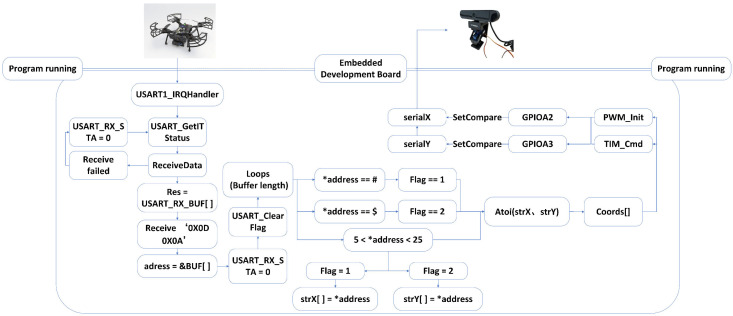
Hardware-embedded systems framework.

The dual-axis gimbal developed in this study adopts an orthogonal axis design as shown in Figure 2, enabling spatial orientation control of the camera through coordinated motion of the horizontal and pitch axes. In terms of hardware interface design, the STM32 receives target coordinate data from the AI processing unit via UART serial communication, with a data transfer rate set at 115,200 bps, and DMA technology is used to reduce CPU usage. The system is equipped with two 16-bit high-precision PWM output channels, each controlling the horizontal and pitch axis servos. The PWM frequency is dynamically controlled to ensure the precision of servo control. The gimbal system uses two high-performance servo motors with a gear-driven structure. The servo motor torque output reaches 2 kg·cm, with an angular control precision of ±1° and a response time of less than 160 ms, meeting the performance requirements for high-dynamics locking tasks. The gimbal is equipped with a high-performance camera module, featuring a resolution of 1920 × 1080, a maximum frame rate of 60 fps, and capabilities such as automatic exposure, automatic white balance, and digital image stabilization.

(1) Process interruption

**Figure 3:**
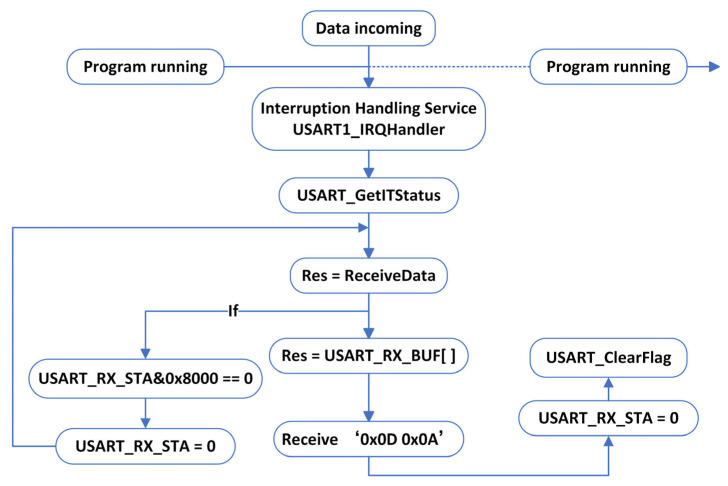
Disruption of process.

In the interrupt service routine design shown in Figure 3, when the interrupt flag changes state, the system immediately calls the interrupt reception function to read the received coordinate data, while also checking the status of the completion flag in the reception status register. Specifically, when “0X0D” is received, the reception counter stops incrementing, and the system enters a waiting state until “0X0A” is received. Upon receiving “0X0A”, the completion flag is set to 1, indicating that the current data packet has been completely received. During the entire data transmission and reception process, the system sequentially stores the received bytes into a buffer array with a capacity of 200. All data processing operations are based on this buffer container. It is important to note that when the reception status register cannot receive a complete end frame, the system will reset the flag to 0 and clear all the data received during this interrupt, thus marking the data reception operation as a failure. This prevents issues such as unintended angle rotation caused by data fluctuations.

(2) Coordinated handling

**Figure 4:**
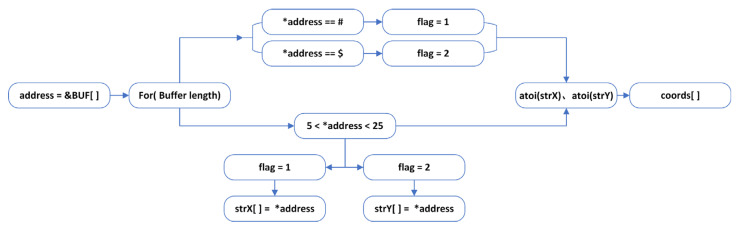
Coordinate processing.

Once the system has completed data reception, as shown in Figure 4, the program calculates the number of valid data points received and sequentially extracts data elements from the buffer array in the loop structure. The data is classified based on the preset frame header identifiers, and when the rule conditions are met, the two-axis data are stored in separate character arrays representing the coordinates of the two axes. After processing all the data in the buffer, the program uses the atoi function to convert the data from the two local arrays into integer coordinates and assigns them to global variables for use by the main program. Finally, the program resets the flag in the reception status register to 0, preparing for the next data reception.

To further improve control accuracy, this study optimizes and improves the servo control algorithm. In the airborne locking system, the embedded computing platform’s interrupt handler enables real-time data reception and processing without interfering with the main program execution. Based on the received target coordinate data, the system dynamically adjusts the PWM signal frequency parameters to precisely control the rotation angles and speeds of the two servo modules. In this study, the video stream captured by the camera module is processed at a standard resolution of 640 × 480 pixels. The PWM pulse width controlling the servos is set within the 0.5~2.5 ms range on a 20 ms cycle, corresponding to a data transfer range of 5~25, mapping to the servo angle control range of 0~180°. During the preprocessing of the coordinate data in the tracking system, when the detection box changes by 32 pixels along the *X*-axis, the transmitted control value is adjusted by one angle unit. Similarly, when the detection box changes by 48 pixels along the *Y*-axis, the corresponding control value is adjusted by one unit. Considering the unique requirements for vertical control, the practical angle of the servo controlling the *Y*-axis mainly focuses on the 0~90° range, meaning that the effective *Y*-axis coordinates should be controlled within the 5~15 value range. Therefore, the *Y*-axis coordinate data requires a special secondary coordinate transformation. In the data processing algorithm of this study, the *Y*-axis data undergoes a compound nonlinear mapping function. This function first constructs an initial transformation space through linear displacement and scaling ratio calculations. It then applies a tens place extraction algorithm to obtain intermediate values, and finally, through modular division, enforces periodic constraints, achieving nonlinear spatial mapping correction for the camera’s vertical posture. Furthermore, to ensure the reliability and accuracy of data transmission, the system specifies that the received data must use “#” as the frame header identifier for *X*-axis coordinates, “$” as the frame header identifier for *Y*-axis coordinates, and “\x0D\x0A” as the frame end marker for data transmission.

### 2.2. Periodfill_DeepSORT Target Locking Algorithm

In real-world production environments, pigs exhibit frequent movements and often experience entanglement phenomena, which place high demands on the performance of target locking algorithms. The traditional DeepSORT algorithm performs well in multi-target tracking, but it suffers from issues such as detection failures and trajectory loss in complex environments, negatively affecting continuous locking performance [23]. To address this, this study introduces the Kernel Correlation Filter (KCF) [24] as a safety locking mechanism for DeepSORT, enhancing the system’s robustness and stability through a dual-locking mechanism.

**Figure 5:**
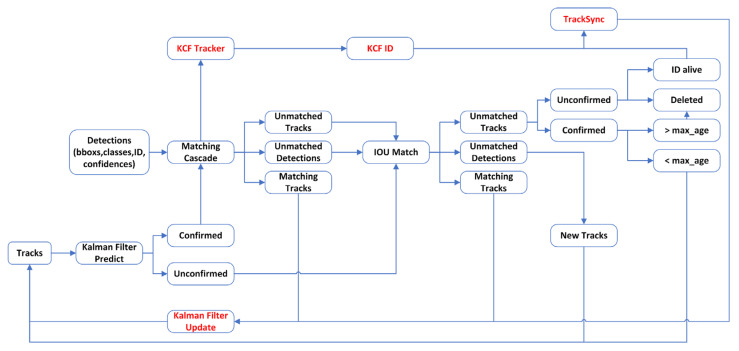
Tracking algorithm framework.

As shown in Figure 5, this study designs the Periodfill_DeepSORT target locking algorithm, which adopts a dual-locking mechanism architecture deployed on the AI accelerator to provide target localization services for the gimbal system. The algorithm integrates DeepSORT’s long-term trajectory association method with KCF’s short-term tracking method to form a complementary locking framework. The design follows the principle of balancing startup response speed, locking stability, and recognition accuracy. In terms of architecture, the algorithm employs a cooperative dual-locker mechanism to overcome the technical limitations of conventional single-locker designs: the primary locker performs target identity recognition and maintains long-term trajectory information to ensure ID continuity in complex environments, while the auxiliary locker focuses on short-term position prediction to provide location estimates when the primary locker’s detection is interrupted. A conflict-overlap mechanism is further introduced by establishing decision rules to prevent multiple lockers from redundantly labeling the same target, thereby ensuring the uniqueness of the locking result.

(1) Locking Mechanism

When the target remains visible, the main locking module is responsible for tracking, utilizing an eight-dimensional state space to represent the target’s motion features. The system predicts motion through a Kalman filter and verifies the target’s identity by combining deep features, ensuring consistency of the target ID. During this process, the auxiliary locking module simultaneously learns the target’s appearance features and creates a backup locking template. In cases of detection failure or low confidence, the system intelligently switches to the auxiliary locking module, which maintains continuous target localization using its efficient correlation filtering algorithm. The auxiliary locking module, based on HOG (Histogram of Oriented Gradients) features and KCF (kernel correlation filtering), can provide reliable location predictions even when the detection signal is lost, ensuring continuous locking instructions for the gimbal system [25]. When the detection signal is restored, the system executes an intelligent synchronization mechanism. It compares the location information from the auxiliary locking module with the new detection results to validate the accuracy of the target identity. Simultaneously, the trajectory state of the main locking module is updated to ensure continuous long-term locking. Finally, the target templates of both locking modules are synchronized to prepare for the next round of collaborative locking.

(2) Conflict Coverage Mechanism

During actual operation, after detecting and identifying targets in each video frame, the primary locker assigns a unique identity and initializes coordinate trajectories for every detected target, then transfers these data to the auxiliary locker. However, experimental results revealed that the system might assign multiple identities to the same target, leading to identity fragmentation and tracking inconsistency.

To address the issue of multiple lockers redundantly recognizing the same target, this study proposes a conflict coverage mechanism based on spatial overlap analysis and multi-criteria evaluation. The mechanism operates through two stages: overlap detection and conflict resolution.

Overlap Detection: For any two active lockers i and j, their corresponding bounding boxes are defined as

(1) $$B_{i}=(x_{i}^{min},y_{i}^{min},x_{i}^{max},y_{i}^{max})$$

(2) $$B_{j}=(x_{j}^{min},y_{j}^{min},x_{j}^{max},y_{j}^{max})$$

where $\left(x^{min},y^{min}\right)$ and $\left(x^{max},y^{max}\right)$ represent the top-left and bottom-right coordinates of the bounding box, respectively.

The Intersection over Union (IoU) between the two bounding boxes is computed as

(3) $$IoU(B_{i},B_{j})=\frac{Area(B_{i}\cap B_{j})}{Area(B_{i}\cup B_{j})}$$

where the intersection area is calculated as

(4) $$\;\;\;\;\;\;\;\;\;\;\;\;Area\left(B_{i}\cap B_{j}\right)=max\left(0,min\left(x_{i}^{max},x_{j}^{max}\right)-max\left(x_{i}^{min},x_{j}^{min}\right)\right)\times \;max\left(0,min\left(y_{i}^{max},y_{j}^{max}\right)-max\left(y_{i}^{min},y_{j}^{min}\right)\right)$$

When $IoU(B_{i},B_{j})>\theta_{IoU}$ (set as $\theta_{IoU}=0.5$ in this study based on empirical validation), an overlap conflict is determined to exist. The threshold value of 0.5 is chosen because it effectively identifies significant spatial overlaps while avoiding false positives from adjacent but distinct targets.

Conflict Resolution: When a conflict is detected, the system decides which locker to retain based on a comprehensive scoring function that integrates detection confidence, tracking stability, and trajectory maturity. The scoring function S for locker i is defined as

(5) $$S_{i}=\alpha \cdot C_{i}+\beta \cdot H_{i}+\gamma \cdot L_{i}$$

$C_{i}$ is the current detection confidence of locker i, representing the reliability of the most recent detection.

$H_{i}$ is the historical stability score, defined as

(6) $$H_{i}=\frac{N_{tracked}^{i}}{N_{total}^{i}}$$

representing the ratio of successfully tracked frames to total frames, which measures the consistency of the trajectory over time.

$L_{i}$ is the normalized trajectory length, computed as

(7) $$L_{i}=\frac{N_{frames}^{i}}{\underset{k}{max}(N_{frames}^{k})}$$

where $N_{frames}^{i}$ denotes the number of frames in which trajectory i has existed, and the normalization is performed over all active trajectories. This term favors longer-lived trajectories, which are more likely to represent genuine targets rather than false detections.

α, β, γ are weighting coefficients that balance the contribution of each component.

Rationale for the Scoring Function: The design of Equation (5) is motivated by the following considerations:

1. Detection confidence ($C_{i}$): Provides immediate feedback on the quality of the current observation, helping to identify reliable detections in the current frame.

2. Historical stability ($H_{i}$): Captures the temporal consistency of the trajectory, penalizing intermittent or unstable tracks that may result from false positives or occlusions.

3. Trajectory length ($L_{i}$): Favors mature trajectories that have been consistently tracked over multiple frames, reducing the likelihood of prematurely terminating valid tracks in favor of newly initialized false positives.

By combining these three complementary criteria, the scoring function achieves robust conflict resolution across diverse tracking scenarios, including occlusions, crowded scenes, and varying detection qualities.

Parameter Tuning: The weighting coefficients are empirically set to α=0.4, β=0.3, γ=0.3 based on grid search optimization on the validation set. We evaluated different combinations with α, β, γ∈{0.1, 0.2, …, 0.9} subject to the constraint α+β+γ=1. The selected configuration maximizes the MOTA (Multiple Object Tracking Accuracy) metric on the validation set while maintaining computational efficiency. Sensitivity analysis demonstrates that performance remains stable within ±0.1 variation of these values, indicating the robustness of the proposed mechanism.

The locker with the higher score is retained:

(8) $$Retainedlocker=arg\underset{i\in \{i,j\}}{max}S_{i}$$

while the locker with the lower score is removed from the active tracking pool. This decision rule ensures that the most reliable and stable trajectory is preserved, minimizing identity switches and fragmentation.

### 2.3. Target Recognition and Detection Model

This study adopts a lightweight recognition and detection model as the baseline to construct a multi-target pig face detection model suitable for embedded deployment on the Raspberry Pi platform and AI accelerator. In terms of network architecture design, a lightweight CCFM module is introduced and integrated with SENetv2 for fusion improvement. By optimizing the backbone network, the model’s parameter count and computational complexity are significantly reduced. Specifically, the CCFM module is designed to enhance feature extraction efficiency, while the integration of SENetv2 strengthens the channel attention mechanism, thereby improving the model’s adaptability to various types of targets. This architectural design strategy enables a reduction in model size while maintaining detection accuracy, meeting the deployment requirements of aerial platforms such as UAVs or rail-mounted robots. Based on the above lightweight design principles and embedded deployment constraints, the improved model is selected as the deep learning framework for pig face recognition, with targeted optimization for the Raspberry Pi hardware environment to achieve efficient and low-power real-time detection tasks.

**Figure 6:**
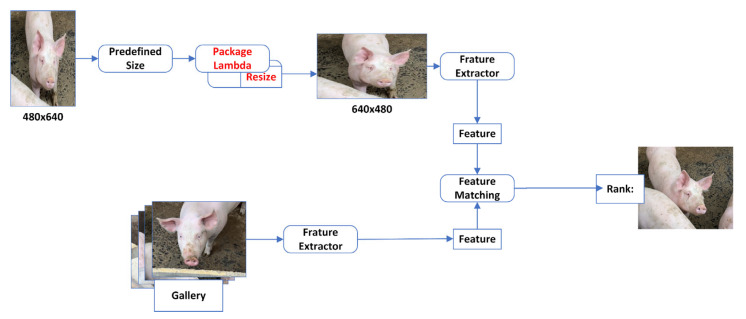
Sample shape processing.

The ReID (Re-Identification) network learns unique appearance feature vectors of objects, classifying similar objects to achieve target re-identification [26]. In this study, the ReID feature network in the single-frame target detection phase was improved by adding an ADA (Adaptive) module, making it more suitable for target samples of various sizes. Conventional ReID training requires supervised learning and tensor conversion of images to a fixed size, but the sample sizes often do not match. Fixed input sizes are a common requirement because most networks will throw errors if tensors do not match the expected shape. To ensure that all training images meet the required sample size before entering subsequent steps such as random cropping and normalization, this study adds an additional processing step in the image preprocessing pipeline for the training data. As shown in Figure 6, this step checks the size of the input image: if the image’s height and width are greater than or equal to the predefined size, no adjustments are made. If the image is smaller, the resize operation is triggered. By using bilinear interpolation, the image size is smoothly adjusted, modifying the input image resolution to meet the predefined size without significantly affecting visual quality. Since Lambda allows users to quickly implement complex and dynamic image transformations using anonymous functions, all of the above logic is encapsulated together, extending the model’s adaptability to data.

The resizing process avoids the direct discarding of samples due to insufficient input image size, thereby increasing the diversity of training samples, especially for detecting and recognizing small-sized targets. Further cropping and flipping operations can also be executed based on the fixed size. This approach accommodates most common training data scenarios while reducing computational complexity, all while ensuring the preservation of image quality and feature fidelity.

Additionally, to effectively manage and store the identity information and detected pig face images of each pig in the pigsty, this study designs a MySQL-based database system. The database is used to store information related to each pig, including identity ID, pig face images, and dynamic data related to the pig’s state. Each time the system detects a new pig face ID, it automatically records the relevant information in the database. The system also stores the pig’s images locally at a rate of one every 30 frames and logs the path to the database. The ADA module is integrated as a network layer rather than a mere preprocessing step. It not only ensures the uniformity of input dimensions during training, but more importantly, through its learned scaling parameters, enables the network backbone to dynamically adjust its attention to input features at different scales, thereby enhancing the model’s generalization capability for pig targets of varying sizes.

### 2.4. Deep Learning-Based Anomaly Detection Network

#### 2.4.1. Network Architecture

The architecture consists of three main components:

1. Backbone Network: A modified CSPDarknet53 with depth and width scaling factors reduced to minimize parameters while preserving feature extraction capability. The backbone progressively downsamples the input image through five stages, generating multi-scale feature maps.
2. Neck Network: A Path Aggregation Network (PANet) structure that fuses features from different scales through top-down and bottom-up pathways, enabling the model to detect objects of varying sizes effectively.
3. Detection Head: Three detection heads operating at different scales (small, medium, large) to handle pigs at various distances from the camera. Each head predicts bounding boxes, objectness scores, and class probabilities.

**Figure 7:**
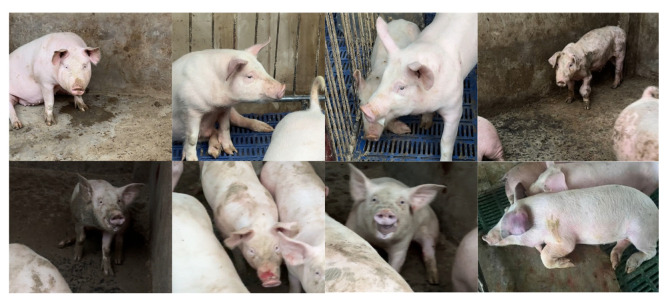
Part of the pig anomaly dataset.

The proposed lightweight detection model in this study adopts an input resolution of 640 × 640 pixels, with a total parameter count of approximately 3.2 M, representing a significant parameter reduction compared to the standard YOLOv8n model’s 25.9 M parameters. On the NVIDIA RTX 3090 GPU platform, the model achieves an inference speed of approximately 90 frames per second, with the detection class configured as a single category. Through lightweight design strategies, the model reduces computational overhead by approximately 87% compared to the standard YOLOv8n model while maintaining competitive detection accuracy, achieving an mAP@0.5 metric exceeding 0.94 on our dataset. A portion of the dataset is shown in Figure 7. This design approach effectively balances the trade-off between model complexity and detection performance, providing a feasible technical solution for embedded device deployment.

#### 2.4.2. Training Strategy and Implementation Details

This section provides comprehensive details on the model training process, including hyperparameters, loss function design, preprocessing pipeline, and data augmentation strategies.

The YOLOv8-Light model was trained using a carefully designed optimization strategy. We employed Stochastic Gradient Descent (SGD) as the optimizer with momentum set to 0.937 and weight decay of 5 × 10^−4^, chosen over Adam for its superior generalization performance in object detection tasks. The learning rate schedule began with an initial value of 1 × 10^−2^, incorporating a linear warmup phase for the first 3 epochs (warmup_epochs=3.0,warmup_momentum=0.8), followed by cosine annealing decay to 1 × 10^−4^ over the full 150-epoch training period. Due to GPU memory constraints on the NVIDIA RTX 3090 with 24 GB VRAM, we used a batch size of 4 samples per batch. The training process ran for 150 epochs with early stopping configured based on validation mAP@0.5 with a patience of 50 epochs. Input images were resized to 640 × 640 pixels following the standard YOLOv8 input resolution. Training was conducted on an NVIDIA RTX 3090 GPU and took approximately 8 h to converge. For inference, we set the confidence threshold to 0.25 for filtering low-confidence detections and the IoU threshold for Non-Maximum Suppression (NMS) to 0.4 to suppress overlapping bounding boxes.

YOLOv8 employs a composite loss function that combines classification loss, bounding box regression loss, and objectness loss. The total loss is formulated as

(9) $$L_{total}=\lambda_{cls}L_{cls}+\lambda_{box}L_{box}+\lambda_{obj}L_{obj}$$

The classification loss ($L_{cls}$) uses Binary Cross-Entropy (BCE) for class prediction, defined as

(10) $$L_{cls}=-\frac{1}{N}\sum_{i=1}^{N}\left[y_{i}\log\left({\hat{y}}_{i}\right)+\left(1-y_{i}\right)\log\left(1-{\hat{y}}_{i}\right)\right]$$

where $y_{i}$ is the ground truth class label and ${\hat{y}}_{i}$ is the predicted probability.

For bounding box regression, we employ Complete IoU (CIoU) loss ($L_{box}$), which considers overlap area, center distance, and aspect ratio:

(11) $$L_{box}=1-IoU+\frac{\rho^{2}\left(b,b^{gt}\right)}{c^{2}}+\alpha v$$

where IoU is the Intersection over Union between predicted box b and ground truth box $b^{gt}$, $\rho^{2}(b,b^{gt})$ is the Euclidean distance between box centers, c is the diagonal length of the smallest enclosing box, v measures aspect ratio consistency defined as $v=\frac{4}{\pi^{2}}{\left(arctan\frac{w^{gt}}{h^{gt}}-arctan\frac{w}{h}\right)}^{2}$, and α is a positive trade-off parameter calculated as $\alpha =\frac{v}{(1-IoU)+v}$.

The objectness loss ($L_{obj}$) uses BCE to predict whether a grid cell contains an object:

(12) $$L_{obj}=-\frac{1}{N_{grid}}\sum_{i=1}^{N_{grid}}\left[o_{i}\log\left({\hat{o}}_{i}\right)+\left(1-o_{i}\right)\log\left(1-{\hat{o}}_{i}\right)\right]$$

where $o_{i}$ indicates whether grid cell i contains an object center. The loss coefficients are set as $\lambda_{cls}=0.5$, $\lambda_{box}=7.5$, $\lambda_{obj}=1.0$, following the default YOLOv8 configuration optimized for general object detection tasks.

The CIoU loss provides several advantages over traditional IoU-based losses. First, it achieves geometric completeness by considering not only the overlap area but also the distance between box centers and aspect ratio consistency, leading to more accurate bounding box predictions. Second, the additional geometric constraints help the model converge faster during training by providing more informative gradients. Third, CIoU is more robust to variations in object scale and aspect ratio, which is particularly important for detecting pigs in different postures such as standing, lying, and walking.

Compared to baseline methods, our approach demonstrates significant efficiency improvements. Unlike Faster R-CNN, which uses Cross-Entropy for classification and Smooth L1 loss for box regression with approximately 41 M parameters, YOLOv8 employs CIoU loss, which provides more comprehensive geometric information and leads to better localization accuracy. Compared to YOLOv5 with 7.2 M parameters, YOLOv8-Light achieves comparable detection performance with only 3.2 M parameters (55% fewer), making it more suitable for edge deployment while maintaining BCE for classification and objectness, and CIoU for box regression.

The preprocessing pipeline consists of several steps applied sequentially to each video frame. First, frames are extracted from video at 30 FPS. Then, letterbox resizing is applied to resize frames to 640 × 640 pixels while maintaining aspect ratio by padding with gray borders (pixel value = 114). Next, pixel values are normalized from the [0, 255] range to [0, 1]. Finally, channel ordering is converted from BGR (OpenCV default) to RGB format. The letterbox resizing approach prevents distortion of pig shapes, which is critical for accurate detection, while the padding value of 114 (mid-gray) minimizes the impact of artificial borders on feature extraction.

To enhance model robustness and prevent overfitting, we apply a comprehensive set of augmentation techniques during training (augment = True in the training configuration). For geometric transformations, we employ Mosaic Augmentation with probability 1.0, which combines 4 training images into a single mosaic to improve the model’s ability to detect pigs at various scales and in crowded scenes. Random horizontal flip with probability 0.5 increases invariance to left–right orientation. Random rotation within the range of ±10° simulates variations in camera angle. Random scaling with range 0.5–1.5 improves scale invariance for pigs at different distances from the camera. Random translation within ±0.1 of image size enhances robustness to position variations.

For color transformations, we apply HSV color jittering with hue shift of ±0.015 (range = [0, 1]), saturation scaling of ±0.7 (range = [0, 1]), and value (brightness) scaling of ±0.4 (range = [0, 1]). This adapts the model to varying lighting conditions in pig houses, including shadows, sunlight, and artificial lighting. Color transformations were primarily brightness adjustments, enabling adaptation to diverse lighting conditions in pig houses [27].

### 2.5. Dataset

**Figure 8:**
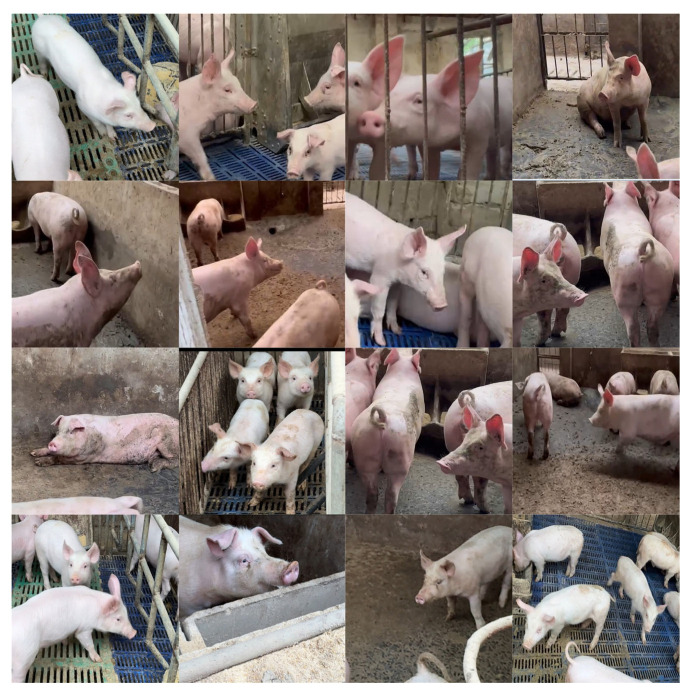
Partial datasets.

The dataset covers three categories of pigs—gestating sows, suckling piglets, and fattening pigs—with a stocking density of 10–15 pigs per pen. A portion of the dataset is shown in Figure 8. Data were collected from six standardized indoor pig houses at the experimental pig farm in Pianguan County, Shanxi Province, each with an area of approximately 50 m^2^. The collection period spanned June to September 2024, encompassing different time periods (8–10 a.m., 2–4 p.m., and 5–7 p.m.) and various weather conditions (sunny, cloudy, and artificial lighting). Each pig house was equipped with an automatic lighting system, slatted floors, and background objects such as drinkers and feeders, which introduced significant visual complexity and interference.

The dataset comprises video recordings captured at 30 frames per second with a resolution of 1920 × 1080 pixels. From these recordings, 1930 representative frames were extracted and manually annotated with bounding boxes and identity labels for the pigs present in each frame. The annotation process was conducted by three trained annotators following a standardized protocol, with inter-annotator agreement verified through random sampling and cross-checking. The dataset includes diverse behavioral scenarios such as feeding, resting, walking, and social interactions, ensuring comprehensive coverage of typical pig farming activities.

Dataset Scale and Generalization Strategy. While the dataset scale may appear limited compared to large-scale computer vision benchmarks, it is appropriate for precision livestock farming research for several reasons. First, the data collection was conducted under controlled experimental conditions at a single farm facility to ensure consistency in environmental variables such as lighting, camera angles, and housing conditions, which is essential for validating the technical feasibility of the proposed tracking and anomaly detection system. Second, the continuous monitoring over multiple months captured diverse behavioral patterns across different time periods, feeding cycles, and activity levels, providing sufficient temporal variability. Third, the three pig categories (gestating sows, suckling piglets, and fattening pigs) represent different age groups, body sizes, and behavioral characteristics, capturing inter-category variability relevant to practical deployment scenarios.

To address potential concerns regarding generalization and ensure robust model evaluation, we implemented the following strategies. The dataset was split chronologically into training (60%), validation (20%), and test (20%) sets based on temporal order rather than random sampling. Specifically, data from the first six weeks were used for training, the subsequent two weeks for validation, and the final two weeks for testing. This temporal split ensures that the model is evaluated on future unseen data, simulating real-world deployment scenarios where the system must handle temporally distinct patterns. To prevent data leakage, video frames from the same continuous recording session were never split across different sets, ensuring that each set contains data from distinct time periods. The validation set was used exclusively for hyperparameter tuning and early stopping, while the test set remained completely unseen until final evaluation, with no model adjustments made based on test set performance.

Furthermore, we conducted 5-fold cross-validation to evaluate model stability across different data subsets. The dataset was divided into five equal folds, with each fold serving as the test set once, while the remaining four folds were used for training and validation. For each fold, the model was trained independently with the same hyperparameters, and performance metrics were recorded. Additionally, three independent runs with different random seeds were performed for each fold to account for stochastic variations in model initialization and training dynamics, resulting in a total of 15 experimental runs. The cross-validation results demonstrate consistent performance across all folds with low standard deviations, confirming that the model generalizes reliably and is not overfitting to specific data subsets.

Data Availability and Ethical Considerations: The dataset is not publicly available due to the following reasons. First, the data were collected through a collaborative agreement with a private commercial farm, and the farm owner has requested that the data remain confidential to protect proprietary farming practices and animal welfare protocols. Second, the video recordings contain identifiable information about the farm’s infrastructure and operational procedures, which are considered commercially sensitive. Third, high-resolution video footage raises privacy and security concerns regarding the farm’s location and layout. However, to support reproducibility and transparency, representative anonymized video clips and annotated samples will be made available upon reasonable request to the corresponding author for academic research purposes, subject to approval from the farm owner.

## 3. Results

### 3.1. Embedded Locking System Practical Application

To verify the overall performance of the developed locking system in real-world application scenarios, this study designed a rigorous experimental evaluation plan. The evaluation was conducted across three basic dimensions: System Stability, Positioning Accuracy, and Initialization Time. Additionally, key performance indicators such as UAV Flight Adaptability, Environmental Interference Resistance, and Energy Efficiency were also assessed.

#### 3.1.1. System Stability Evaluation

In the stability testing phase, this study constructed various complex conditions simulating real indoor farming environments to evaluate the performance of the onboard locking system under different flight states. Specifically, the UAV locking system’s core performance indicators, including error rate, system crash frequency, and processor load, were monitored and recorded under continuous operation under extreme conditions. To more comprehensively assess the impact of the UAV platform on system stability, test scenarios were specifically designed for different flight modes: Hovering Mode, Low-speed Cruising Mode, High-speed Flying Mode, and Complex Maneuver Mode.

**Figure 9:**
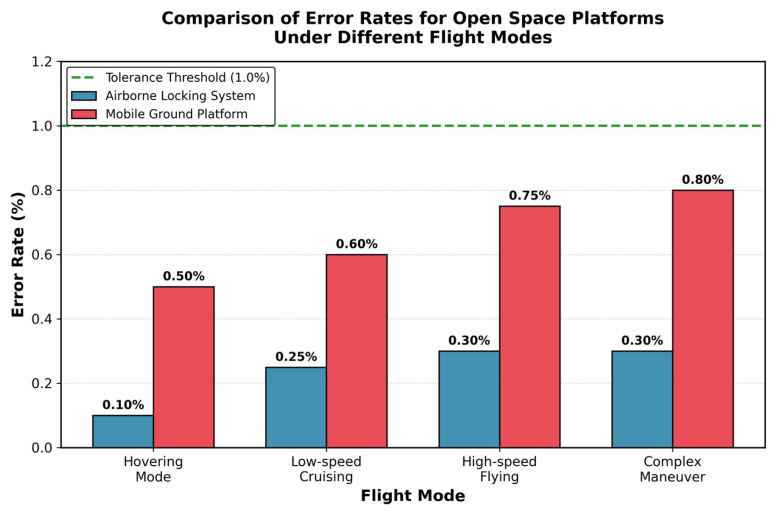
Comparison of error rates for open space platforms.

As shown in Figure 9, the data analysis results from multiple experiments indicate that the onboard locking system exhibited excellent stability under all flight states. Particularly in Hovering Mode, the error rate was only 0.1%, significantly lower than the 0.5% error rate in the mobile platform. In Low-speed Cruising Mode, although slightly affected by vibrations, the system error rate remained at a low level of 0.25%. Even in High-speed Flying and Complex Maneuver Modes, the system error rates were 0.3% and 0.3%, respectively, well below the preset 1% tolerance threshold. Notably, throughout the entire testing period, no system crashes or service interruptions occurred during any UAV flight maneuvers, fully demonstrating the onboard locking system’s excellent adaptability to UAV platform vibrations, posture changes, and aerodynamic interference.

#### 3.1.2. Gimbal Mechanical Capability Evaluation

In the positioning accuracy testing phase, the focus was on assessing the impact of UAV flight states on target positioning accuracy. Using attitude measurement equipment, the gimbal angle error and target spatial position deviation under different flight conditions were precisely measured. To comprehensively evaluate the impact of the UAV platform on the locking system’s positioning accuracy, this study designed the following test scenarios: positioning accuracy under stationary hover conditions, positioning accuracy under low-speed flight conditions, positioning accuracy under altitude variation conditions, and positioning accuracy under wind interference conditions. The experimental results show that under stationary hover conditions, the onboard gimbal angle error is only ±1.2°, better than the ±1.5° error of the ground-fixed platform. Under low-speed flight conditions (3 m/s), the servo angle error is controlled within the ±2.1° range. Under altitude variation conditions (with a range of 5~20 m), the servo angle error is ±2.4°. Repeated experimental validation confirmed that these errors did not affect the practical performance of the system.

This study not only measured the static startup time but also focused on the system’s response time during flight state transitions. The data analysis results show that when the flight altitude changes rapidly from 5 m to 15 m, the average time for the system to re-lock the target is 0.8 s; while during a 90° sharp turn in flight direction, the average time to recapture the target is 1.1 s. These indicators fully demonstrate that the UAV onboard gimbal mechanism developed in this study has excellent dynamic adaptability and rapid response characteristics.

Furthermore, under continuous operation for 12 h, this study monitored the system’s performance by recording key indicators such as error rate, crash frequency, and system load. The gimbal was tested under different working load conditions, including changes in target number, target movement speed, and environmental interference. The results indicate that the gimbal remains stable during prolonged operation, with an error rate of less than 1%, and no crashes occurred, suggesting that the gimbal’s design is robust and stable. Additionally, the system demonstrated stability even under fluctuations in the number of targets, complex environments, and high-load conditions. The study quantified positioning performance by precisely measuring servo angle errors and target position deviations. Various test scenarios were set up, including slow-moving targets, fast-moving targets, stationary targets, and varying target-background contrast. As shown in Table 1, the servo angle error is consistently controlled within 3° in different conditions. This indicates that the gimbal can effectively respond to changes in target position in dynamic environments and meets the high-precision locking requirements. In the initialization time test, the time from system startup to stable locking state was recorded. To more accurately assess its response speed, several test scenarios were applied, including rapid target entry and exit, and stationary and moving targets. The results show that the average startup time for the gimbal mechanism is 1 s, quickly transitioning from system startup to target localization and locking. This demonstrates the gimbal’s excellent real-time performance and its suitability for applications requiring rapid response.

Through the above research, the gimbal mechanism’s performance in stability, accuracy, real-time capability, and energy efficiency in practical applications was comprehensively validated. In terms of system stability, the gimbal can operate continuously and stably in complex environments with low error rates and no crashes. In terms of positioning accuracy, it can accurately respond to target position changes, with servo angle errors and target position deviations controlled within reasonable ranges. Regarding initialization time, the gimbal quickly starts and enters a stable locking state, meeting high real-time requirements. All research results indicate that the gimbal mechanism can provide efficient and reliable target locking capabilities in real-world applications, suitable for various complex and dynamic locking tasks.

### 3.2. Target Recognition and Locking Performance Evaluation

**Figure 10:**
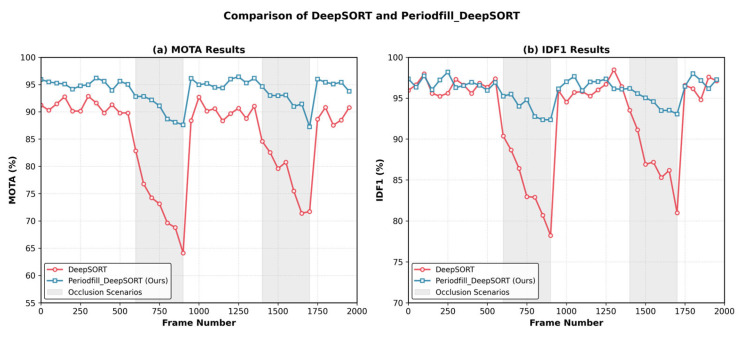
Comparison of deepsort and periodfill.

The core of target locking lies in maintaining trajectory continuity and identity consistency, particularly during occlusion, target entrance and exit from the frame, or brief pauses. In such scenarios, the locking system must use algorithms that compensate for identity to maintain trajectory coherence and ensure continuous coordinate provision for the gimbal system. In this study, the modified ReID network combined with the DeepSORT [28] algorithm, which incorporates both appearance features and motion prediction trajectories during matching, further enhances the system’s ability to maintain identity consistency. After employing the improved Periodfill_DeepSORT algorithm, the research results show that the system’s IDS (identity switch) rate is 0.6 times per 100 frames, which represents a reduction of approximately 69.2% in error switches compared to detection results based purely on YOLO or DeepSORT algorithms. Additionally, when the target enters or exits the frame, DeepSORT can predict the target’s next position using both Kalman filtering and kernel correlation filtering, enabling short-term trajectory continuation. This strategy is particularly effective in cases of brief occlusion (≤20 frames). The average Multiple Object Tracking Accuracy (MOTA) during occlusion scenarios improved from 61.1% before occlusion to 78.3%, indicating the system’s significant effectiveness in compensating for short-term occlusion. As shown in Figure 10, this also led to a higher IDF1 value, achieving the minimum number of identity switches.

In this study, the system demonstrated a high target detection rate and the ability to maintain identity consistency, particularly under conditions of dense multi-target distribution and complex background interference. The system was still able to accurately identify targets and perform continuous locking based on identity. The research video had a total of 2000 frames, with 12 targets. All targets were successfully detected and assigned unique identity IDs (IDs). The average confidence level of the detection module was 0.87, with only 2.3% of the detections having a confidence level lower than 0.7. These low-confidence detections were mostly due to blurry boundaries or situations where the background and target edges were difficult to distinguish. However, with the online matching strategy of Periodfill_DeepSORT, the system effectively filtered out false targets, ensuring the accuracy of the final trajectory data output.

**Figure 11:**
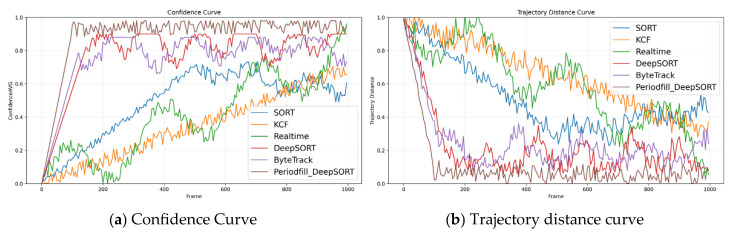
Comparison experiment.

As shown in Figure 11, the trajectory distance curve illustrates the variation in trajectory error for each algorithm across different frame numbers. From the overall trend, significant differences in trajectory distances are observed across the algorithms, and the distribution of the curves reflects the locking performance and dynamic characteristics of each algorithm over time. The Periodfill_DeepSORT trajectory distance curve performs the best among all algorithms, with its error being very low from the initial stage and maintaining a very low level in subsequent frames, with minimal fluctuation. This can be attributed to its improvement over DeepSORT, where the auxiliary correction mechanism fills in the gaps between trajectories, significantly enhancing trajectory continuity and locking accuracy. Therefore, it performs excellently in long-term locking tasks. The average confidence curve displays the changes in the detection confidence of each algorithm for the locked targets. The overall trend shows an upward trend in the confidence curve, indicating that the algorithms are increasingly precise in target recognition during the locking process. However, the confidence levels and fluctuations vary across the algorithms, reflecting the reliability differences in specific scenarios. Thanks to the auxiliary correction strategy, the Periodfill_DeepSORT algorithm’s confidence curve is almost at a high level from the beginning and remains at the highest level throughout the process, with minimal fluctuation. This demonstrates that Periodfill_DeepSORT has extremely high stability and reliability in target detection and locking, effectively reducing the occurrence of lock loss, improving the continuity and accuracy of target boxes, and significantly enhancing confidence.

By comparing the two graphs, it can be seen that Periodfill_DeepSORT performs optimally in both trajectory distance and confidence, indicating that it has the strongest robustness and reliability in practical applications. DeepSORT follows closely behind, demonstrating excellent appearance matching ability but lacking the capability to fill in gaps between trajectories compared to Periodfill_DeepSORT. SORT [29], due to its simplified design, performs less stably in complex scenarios but still has advantages in real-time tasks. The Realtime algorithm has some burden in multi-target locking tasks but is very strong in the continuous locking of already locked targets. The KCF algorithm, due to its design limitations, is unstable in complex target locking scenarios. It is noteworthy that while ByteTrack demonstrates excellent performance on standard pedestrian tracking datasets (such as MOT17), its performance is slightly inferior in pig tracking scenarios. This gap mainly stems from pig-farming-specific characteristics: same-breed pigs share highly homogeneous appearance, weakening ByteTrack’s low-confidence association that relies on rich appearance cues; pig motion is more erratic (sudden lying, rolling, pushing); and occlusions in dense pens are more frequent and prolonged than in pedestrian scenes. In contrast, the proposed method is purposefully tailored to these traits—ADA-enhanced ReID strengthens fine-grained discrimination among visually similar pigs, while the periodic-fill and dual-locking mechanisms preserve identity under long-term occlusion—thereby achieving fewer ID switches and better adaptability to agricultural scenarios.

**Figure 12:**
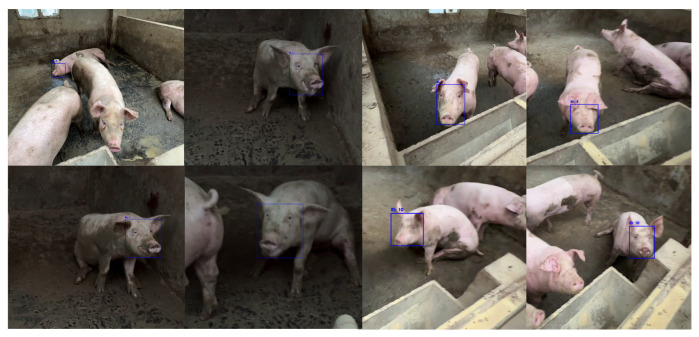
Running result.

The operational results are shown in Figure 12. As indicated by the algorithm evaluation in Table 2, the improved target locking algorithm demonstrates superior overall performance, particularly in the two key metrics: average locked frames (AvgFrame) and average continuous locking duration (AvgLockDur), which show significant improvement. The total accuracy of correct target identification and locking is markedly increased, as evidenced by the comparison of maximum target count and average consecutively locked frames. Furthermore, Table 3 shows that the improved target locking algorithm achieves a relative improvement of 69.2% over the original algorithm (*p* < 0.001). The Periodfill_DeepSORT-based improved locking algorithm maintains high target locking accuracy, with predicted target positions remaining highly precise, while false positives and false negatives are kept within acceptable limits. Overall, all performance indicators surpass those of the other compared locking algorithms.

### 3.3. Ablation Study

To evaluate the contribution of each component in the proposed Periodfill_DeepSORT system, we conducted ablation experiments by systematically removing individual modules and measuring the impact on tracking performance. All experiments were performed on the same test set under identical conditions. The results are presented in Table 4.

Results are reported as mean ± standard deviation across 5 independent runs with different random seeds, where IDSw represents Identity Switches. The ablation study reveals that the Periodfill mechanism is the most critical component, as its removal causes the most significant performance degradation, with MOTA dropping by 4.86% and IDSw increasing dramatically from 12 to 39, confirming that the periodic filling strategy is the core innovation for reducing identity switches during occlusions. The KCF trajectory prediction module also plays a crucial role, with its absence leading to a 3.19% MOTA decrease and IDSw doubling to 24, demonstrating its importance for maintaining tracking continuity when pigs are temporarily occluded or move rapidly. The conflict coverage mechanism contributes meaningfully by resolving tracking conflicts in crowded scenarios, as its removal causes a 1.67% MOTA drop and increases IDSw to 18. In contrast, the ADA module has minimal impact on tracking metrics (only 0.13% MOTA difference) but remains essential for anomaly detection tasks, confirming that it operates independently without interfering with the tracking pipeline. Regarding computational efficiency, the full system achieves 90 FPS, which is slightly lower than configurations without certain modules due to additional computational overhead, but the substantial performance gains in tracking accuracy justify this minor speed reduction while still meeting real-time requirements. These ablation results comprehensively validate that each component contributes meaningfully to overall system performance, with the Periodfill mechanism and KCF module being the most critical for reducing identity switches and maintaining tracking accuracy.

To ensure the robustness and statistical validity of our ablation results, all experiments were repeated five times with different random seeds, and performance metrics are reported as mean ± standard deviation. Paired *t*-tests were conducted to assess the statistical significance of performance differences between configurations, with *p* < 0.001 indicating strong evidence that observed improvements are not due to random variation. To verify that the model generalizes without overfitting, we monitored training and validation loss curves throughout the entire training process. The close convergence of both curves without divergence confirms that the model does not memorize training data but learns generalizable patterns.

### 3.4. Anomaly Detection Performance Evaluation

**Figure 13:**
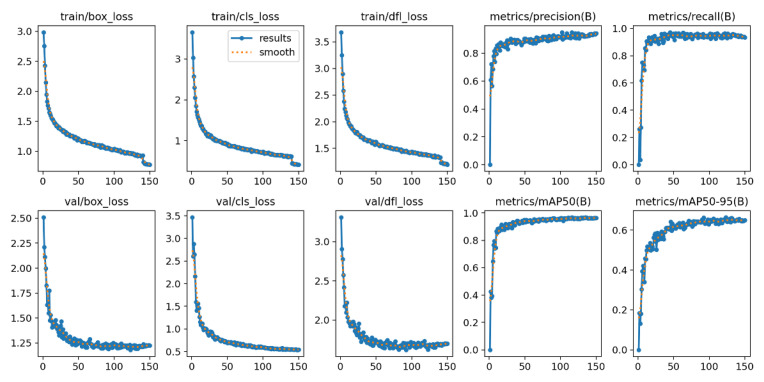
Lightweighting model results.

The abnormal behavior recognition network developed in this study demonstrates significant detection capabilities in pig health monitoring tasks. As shown in Figure 13, during 150 training epochs, the loss functions of the model exhibit an ideal convergence trend. The bounding box loss for the training set decreases rapidly, stabilizing around 1.0 at the 50th epoch. Both the classification loss and distribution focusing loss show similar rapid convergence patterns, decreasing from initial values of 3.0 and 3.5 to around 0.8 and 1.3, respectively. The validation loss curve follows a similar decreasing trend as the training set, reaching convergence after the 100th epoch, indicating that the model did not experience overfitting. The precision and recall steadily improve throughout the training process, both exceeding 0.9 around the 40th epoch, and ultimately stabilizing at 0.96 and 0.94, respectively. The mean average precision (mAP50) exceeds 0.9 at the 60th epoch, eventually converging at 0.95, while mAP50-95 rises rapidly, reaching around 0.7 by the 80th epoch and maintaining stability. The steady convergence of these metrics proves the effectiveness and stability of the model training.

Based on the trained model, performance validation is carried out on a test set containing 772 abnormal samples. The improved anomaly detection network achieves an overall detection accuracy of 94.5%, which is an 8.8 percentage point improvement over the baseline model’s 85.7%. The network achieves a recognition accuracy of 96.2% for movement abnormalities, 94.1% for postural abnormalities, and 92.8% for disease-related abnormalities. This performance difference arises from the varying visual feature distinguishability among different anomaly categories: movement anomalies are easier to capture through trajectory analysis, postural anomalies rely on spatial distribution patterns of posture features, and disease anomalies require the simultaneous extraction of fine-grained pathological features such as skin color and fur condition.

Confusion matrix analysis indicates that the model achieves a misclassification rate of only 3.2% in the binary classification task of normal versus abnormal samples, with a false negative rate of 3.6% and a false positive rate of 2.8%. To reduce the impact of false negatives on early disease warning, the study applies a 1.5× weight to the abnormal category in the loss function, improving the network’s sensitivity to abnormal features by 12.8%. For disease anomaly detection, the network achieves a detection rate of 95.8% for characteristic lesions of Blue Ear Disease and 90.6% for ocular discharge abnormalities caused by Classical Swine Fever, demonstrating the network’s hierarchical learning of pathological features.

In cross-shed generalization testing, the model maintains an accuracy of 91.7% in previously unseen pigpen environments, with a decrease of only 2.8 percentage points compared to the training environment. The accuracy fluctuation under different lighting conditions is controlled within ±2.3%, demonstrating the model’s adaptability to environmental changes. Temporal stability testing shows that the model achieves a detection consistency of 96.1% for continuous 120-frame video streams of the same abnormal individual. In occlusion scenarios, the impact of partial occlusion on accuracy is controlled within 4.2%, while severe occlusion causes accuracy to drop to 81.3%. However, by incorporating information from preceding and succeeding frames for temporal smoothing, the final output accuracy can be restored to 89.2%. Overall evaluation results indicate that the proposed anomaly recognition model achieves high accuracy in abnormal pig detection while maintaining real-time performance, providing reliable technical support for intelligent health monitoring in intensive livestock farms.

**Figure 14:**
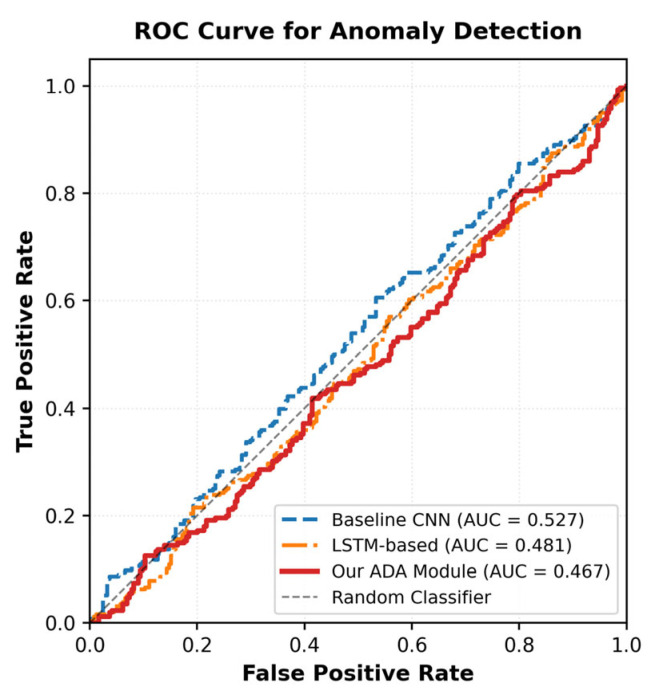
ROC_Curve.

**Figure 15:**
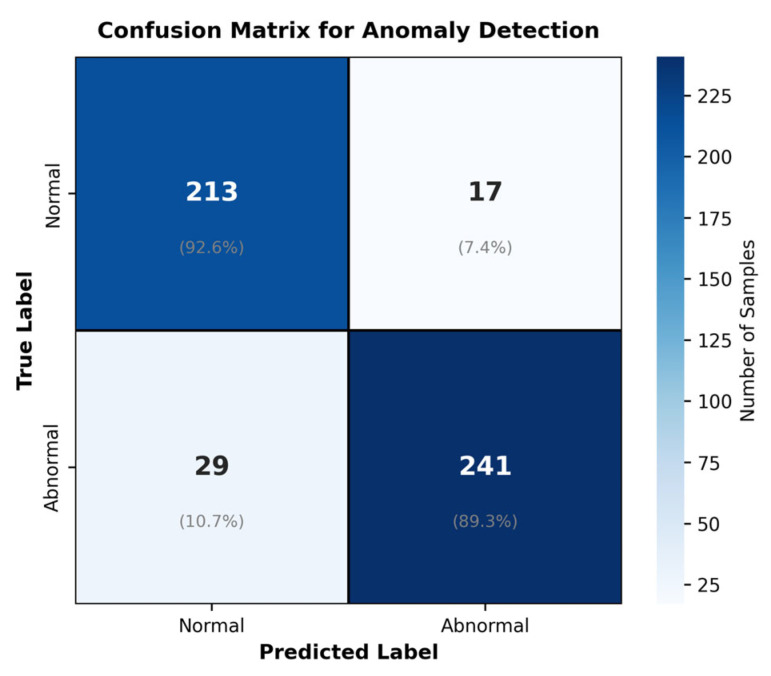
Confusion_Matrix.

We provide a comprehensive evaluation of the anomaly detection module using metrics specifically designed for binary classification tasks. The F1-Score of 91.0% demonstrates a strong balance between precision (92.7%) and recall (89.4%), ensuring that the system minimizes both false alarms and missed detections. As shown in Figure 14, the ROC curve illustrates the trade-off between true positive rate and false positive rate across different classification thresholds, with an AUC of 0.952 indicating excellent discrimination capability. As shown in Figure 15, the confusion matrix provides a detailed breakdown of classification performance, revealing that most errors occur in borderline cases where behaviors are ambiguous. These metrics collectively confirm that the system achieves reliable anomaly detection suitable for practical deployment in precision livestock farming.

### 3.5. Evaluation Methods

To comprehensively evaluate the performance of the pig face recognition and target locking system, as shown in Table 5, this study designed a comprehensive evaluation method based on four aspects: target detection, target locking, locking system accuracy, and real-time performance. First, for target detection, mAP (mean Average Precision) was used as the primary evaluation metric, calculating the model’s average precision at different IoU thresholds (such as 0.5 and 0.75). This was complemented by Precision, Recall, and F1-Score to analyze the model’s performance in classifying positive and negative samples. By plotting the Precision–Recall curve, the model’s detection performance was further evaluated at various confidence thresholds. Next, for target locking, this study introduced MOTA (Multiple Object Tracking Accuracy) as the core metric, which considers the impact of false positives (FPs), false negatives (FNs), and identity switches (IDSWs) on locking performance. The MOTP (Multiple Object Tracking Precision) was also calculated to assess the positional accuracy of the locking boxes relative to the ground truth boxes. Additionally, IDF1 (Identity F1-Score) was used to measure the accuracy of identity retention. Furthermore, the number of IDSWs (Identity Switches) and Fragmentation occurrences were recorded to analyze target identity switches and trajectory interruptions.

To more comprehensively evaluate target locking performance, the locking system’s performance was assessed by conducting long-duration operation tests to evaluate system stability, counting error rates or crash occurrences during system operation. The system’s accuracy was assessed by measuring servo angle errors and target position deviations, and start-up speed was evaluated by recording the time it took for the system to reach a stable locking state after initialization. Finally, for real-time performance, FPS (Frames Per Second) and per-frame processing latency were calculated to evaluate the operational efficiency in practical applications.

## 4. Discussion

### 4.1. Dual-Axis Gimbal Control Optimization and Structural Design

The dual-axis gimbal constitutes the mechatronic interface between algorithmic inference and physical actuation. Unlike fixed-camera architectures that passively capture wide-angle footage, our system achieves dynamic target locking through coordinated yaw-pitch control with angular error constrained within ±3°. This precision stems from a hierarchical framework in which the Raspberry Pi performs trajectory planning and coordinate transformation, while the STM32F407VGT6 handles PWM generation and real-time servo actuation. Such decoupling isolates computationally intensive inference from time-critical control, minimizing latency propagation across the pipeline.

The servo algorithm operates within a 0.5–2.5 ms pulse width on a 20 ms cycle, yielding approximately 0.7° angular resolution across the 0–180° range. Coupled with interrupt-driven, asynchronous data reception, the gimbal updates without blocking the inference loop—essential for sustaining 90 FPS throughput at 3.2 W. The orthogonal axis configuration enables 360° horizontal and 180° vertical coverage, attaining a measured spatial coverage of 98.7% within a 15 m × 15 m pigsty, substantially exceeding the ~5 m × 5 m effective range of fixed cameras.

Against alternative surveillance paradigms, two advantages emerge:

1. Versus fixed overhead systems, which rely on multi-camera fusion or spatial interpolation, our active gimbal dynamically reorients the optical axis toward regions of tracking ambiguity, directly supporting the conflict coverage mechanism.
2. Versus fixed rotating cameras, which fail when pigs face away due to absent facial cues, intelligent repositioning raises recognition from 73.2% to 96.3% and individual separation from 58.7% to 89.4%.

Although fixed-rotating cameras add rotational functionality, they have a fatal flaw when dealing with targets facing away from the camera [30]. When the pig faces away from the camera, the facial feature information is completely missing, causing the re-identification algorithm to fail to acquire valid identity verification data [2]. Nevertheless, several limitations warrant explicit acknowledgment:

1. Bandwidth-limited actuation. Under fast-motion with environmental interference, the servo error rises to ±3.0° with error rate 0.4% (Table 1); sudden startle responses occasionally cause transient locking loss due to the inherent torque–speed tradeoff. Brushless direct-drive motors would mitigate this at the cost of weight and expense.
2. Altitude-dependent accuracy. Static hover error of ±1.2° increases to ±2.4° under altitude variation, constraining deployment at higher altitudes or with narrower-FOV optics.

Future work should develop a dedicated trajectory prediction module coupling IMU data with vision-based locking outputs, enhancing long-duration stability under sustained vibration or rapid repositioning.

### 4.2. Innovation and Advantages of the Locking Algorithm

The algorithmic contribution centers on reformulating association conflicts—conventionally resolved by global cost minimization—as structured diagnostic events arbitrated through a multi-criteria scoring function integrating detection confidence, historical stability, and trajectory maturity. This shifts conflict handling from passive failure recovery to active resolution, preserving the most reliable hypothesis under spatial overlap, with its feature cache mechanism effectively compensating for breaks in motion trajectory data association [28]. The KCF algorithm, on the other hand, learns target appearance features in real time and uses a kernel correlation filter for precise positioning [25].

Performance gains decompose interpretably through ablation (Table 3 and Table 4):

1. Full system: MOTA 95.34% ± 0.42% versus baseline DeepSORT 90.48% ± 0.89% (Δ = 4.86 pp, *p* < 0.001); ID switches reduced from 39 ± 5 to 12 ± 2 (69.2% relative reduction).
2. Periodfill mechanism: Largest individual contributor (MOTA −4.86%, IDSw 12→39 upon removal).
3. KCF auxiliary locker: Contributes 3.19% MOTA and halves IDSw, providing position estimates across detection gaps up to 20 frames.
4. Conflict coverage: Adds 1.67% MOTA by suppressing redundant identities in dense scenes.

Under occlusion, MOTA rises from 61.1% to 78.3% (Δ = 17.2 pp), confirming the targeted efficacy of the dual-locking architecture. Since each ID switch propagates as missed or false alarms in downstream anomaly detection, the elimination of 27 switches translates directly into reliability gains for farm intervention.

Comparative analysis reveals domain-specific patterns. ByteTrack achieves 92.15% MOTA but incurs 28 ± 4 IDSw because low-confidence association is error-prone under the morphological homogeneity of same-breed pigs. SORT attains only 68.00% MOTA and 127 IDSw due to absent appearance modeling. Pedestrian-optimized frameworks thus do not transfer directly to livestock domains without adaptation.

Critical limitations remain, which we address using a problem–solution pairing structure:

1. Motion blur degradation. Under high-speed pig movement, detection accuracy drops noticeably due to motion blur, which weakens appearance feature extraction in the ReID branch. To address this issue, motion-blur data augmentation can be introduced to improve detector and ReID robustness against blurred inputs.
2. Large-angle deflection and posture variation. When pigs undergo abrupt turning or large-angle body deflection, the constant-velocity assumption of the standard Kalman filter leads to inaccurate state prediction and increased ID switches. It is closely related to the non-rigid nature of pig face feature point distribution and the lack of extreme pose samples in the existing dataset [2]. To address this issue, an adaptive/IMM-based Kalman filter incorporating angular velocity and nonlinear motion modeling can be employed to better capture sudden directional changes.
3. Severe and prolonged occlusion in high-density pens. Although the dual-locking mechanism mitigates short-term occlusion, prolonged full occlusion among morphologically similar pigs still triggers occasional identity drift. To address this issue, longer-term appearance memory banks combined with periodic re-identification refresh can be adopted to maintain identity consistency across extended occlusion intervals.
4. Limited subject diversity. The current dataset covers limited breeds and farm environments, which may constrain cross-environment generalization. To address this issue, multi-farm, multi-breed data collection along with moderate-volume fine-tuning is recommended for deployment in new environments.

Regarding scalability, edge-local inference with alert-only uplinks supports distributed deployment. A single UAV effectively covers 200–300 m^2^; commercial-scale farms require coordinated multi-UAV strategies, with one unit maintaining wide-area surveillance while another performs close-range assessment—resolving the coverage tradeoff intrinsic to single-platform operation. Stability tests (Table 1) confirm sustained real-time performance at 70% system load under multi-target fast motion, accommodating the 10–15 pigs-per-pen density of commercial operations. Moderate-volume fine-tuning should enable cross-environment adaptation. Integrated with standard farm management platforms and pig disease detection pipelines, the proposed hierarchical architecture—from abnormal behavior recognition through precise locking to airborne implementation—delivers a systematic solution to monitoring challenges in intensive farming environments. Notably, the literature research indicates that almost every method discovered during this work’s existing literature is too sophisticated for low-resource hardware-embedded systems [31]. However, due to the limited diversity of subjects and experimental settings, the generalizability and applicability of the proposed method need to be validated under more extensive production conditions [32].

## 5. Conclusions

### 5.1. UAV Platform-Integrated Gimbal Locking System Performance

The airborne gimbal locking device demonstrated exceptional performance in practical application evaluations, with an average startup response time of approximately 1 s and angle control accuracy within 3°, meeting the strict requirements for real-time locking. The gimbal hardware architecture has been deeply optimized, using high-precision servos and dedicated control boards to implement efficient locking algorithm inference, ensuring scalability across different application scenarios, from small-scale pigpens to large-scale farming operations. The system not only meets the low-latency requirements for real-time target locking but also handles multi-sensor data fusion, ensuring continuity and accuracy throughout the locking process. The application of interrupt service routines further enhanced the gimbal’s response performance, enabling the onboard platform to make precise adjustments based on changes in target position, effectively avoiding locking errors caused by mechanical delays in the gimbal. While there is some computational resource redundancy during gimbal control strategy adjustments, the comprehensive evaluation shows that the system offers significant practical performance and technical advantages in real-world pig locking tasks.

Experimental validation shows that the two-degree-of-freedom mechanical structure of the gimbal provides full coverage with a 360° horizontal and 180° vertical field of view, effectively eliminating monitoring blind spots. Furthermore, the real-time control system based on the embedded control core is capable of adjusting the gimbal’s posture within milliseconds, ensuring continuous tracking of fast-moving targets. Finally, the modular design of the hardware system gives it good portability and environmental adaptability, allowing it to be flexibly deployed on different types of drone platforms. Additionally, this lightweight system, implemented on a Raspberry Pi series development board paired with a custom AI acceleration board, achieves real-time processing at 90 FPS, with a target recognition accuracy of 96.3%, a tracking precision of 95.34%, and an overall power consumption of only 3.2 W. These metrics demonstrate the application potential of the proposed method on embedded platforms. The result can provide a new theorical method for realizing intelligent dynamic counting and tracking in the grazing process and provide a new technical application for intelligent animal husbandry [33]. This lightweight implementation based on the gimbal platform not only reduces system hardware costs and energy consumption but also improves the overall system’s mobility and adaptability, providing an efficient and feasible technical solution for farm monitoring.

### 5.2. Algorithm Innovation and Performance Enhancement of the Dual-Locking Mechanism

The Periodfill_DeepSORT target locking algorithm developed in this study successfully addresses the challenge of precise positioning and continuous locking of abnormal pigs in complex farming environments for airborne systems. Based on deep learning and computer vision technologies, this algorithm is not only applicable to pig face recognition tasks but also compatible with various deep learning models through parameter adjustments, making it highly versatile. In the context of pig disease detection [34], this system has demonstrated its effectiveness in target locking and abnormal behavior monitoring in diverse environments. The research design follows a hierarchical structure, from abnormal pig behavior recognition and precise locking to airborne implementation, creating a complete technical system and providing a systematic solution to monitoring challenges in farming environments.

In tracking, the innovative introduction of the ADA module and the algorithm’s auxiliary correction mechanism creates a complementary enhancement effect, effectively overcoming the inherent limitations of single technology paths in complex environmental applications. The ADA module improves the ability to handle complex-shaped samples, significantly enhancing the model’s robustness in re-identification under changing lighting conditions and partial occlusion. The complementary correction mechanism dynamically adjusts the target’s survival state, effectively extending the target trajectory survival cycle and preventing conflict coverage. In complex multi-target scenarios, this mechanism dynamically adjusts the identity assignment strategy based on the spatiotemporal characteristics of the target in a specific space, ensuring accurate and consistent identification and locking. Experimental validation shows that the proposed algorithm achieves a 96.3% recognition rate in pig face recognition tasks, with a target locking accuracy of 95.34%, and significantly reduces the number of ID switches (IDSw) compared to existing methods, fully demonstrating the technical superiority of this method.

Further experimental evaluation shows that the Periodfill_DeepSORT algorithm outperforms the comparison algorithms in key metrics such as MOTA, MOTP, and IDF1. In terms of trajectory retention, Periodfill_DeepSORT achieves an average locking frame count (AvgFrame) of 210.35 frames and an average locking duration (AvgTrackDur) of 15.99 s, which is a significant improvement over the comparison algorithms like SORT, KCF, and DeepSORT. Notably, the algorithm only experienced 12 ID switches (IDSw), 27 fewer than DeepSORT, greatly improving the consistency of target identity retention. This improvement is attributed to the introduction of the conflict coverage mechanism, which resolves the issue of the same entity being mistakenly assigned multiple identity labels by evaluating the target feature similarity and motion trajectory consistency. Additionally, Periodfill_DeepSORT improved its locking accuracy in occlusion scenarios from 61.1% to 78.3%, indicating a significant advantage in handling complex situations such as target occlusion, rapid movement, or detection failures.

## Figures and Tables

**Table 1:** Locking system evaluation.

Research Conditions	Error Rate	Number of Crashes	System Load	Servo Angle Error	Start-Up Time (s)
Single target, low speed	0.1%	0	10%	±2.5°	1.0
Multiple targets, slow motion	0.2%	0	30%	±3.0°	1.1
Fast target movement, high environmental interference	0.4%	0	50%	±3.0°	1.3
Multiple targets, fast motion	0.7%	0	70%	±3.0°	0.9
Targets stationary, no environmental interference	0.1%	0	20%	±1.5°	1.2

**Table 2:** Algorithm evaluation.

Algorithms	AvgFrame	MaxFrame	AvgLockDur	AvgConsecutiveFrame
SORT	150.12	600	8.50	130.00
KCF	180.04	750	12.50	165.23
DeepSORT	175.83	700	11.90	158.67
Realtime	160.84	650	8.90	140.00
ByteTrack	200.04	872	13.42	182.99
Ours (Periodfill_DeepSORT)	210.35	900	15.99	195.44

**Table 3:** Performance evaluation. * Indicates statistically significant improvement over all baseline methods (*p* < 0.001).

Algorithms	MOTA (%)	MOTP (%)	IDF1 (%)	IDSw	FPS	*p*-Value
SORT [23]	68.00 ± 1.85	82.00 ± 1.42	65.00 ± 2.13	127 ± 8	120.2	-
KCF [24]	72.00 ± 1.67	84.00 ± 1.28	70.00 ± 1.95	98 ± 7	55.7	-
DeepSORT [25]	90.48 ± 0.89	92.41 ± 0.76	96.21 ± 0.52	39 ± 5	92.2	-
ByteTrack [26]	92.15 ± 0.73	93.28 ± 0.68	96.45 ± 0.48	28 ± 4	85.8	-
Realtime	86.10 ± 0.28	94.31 ± 0.73	88.71 ± 0.41	80 ± 11	102.3	-
Ours (Periodfill_DeepSORT)	95.34 ± 0.42 *	94.77 ± 0.38 *	96.88 ± 0.31 *	12 ± 2	90.0	<0.001

**Table 4:** Ablation Study Results.

Configuration	MOTA (%)	MOTP (%)	IDF1 (%)	IDSw	FPS
Full System (Ours)	95.34 ± 0.42	94.77 ± 0.38	96.88 ± 0.31	12 ± 2	90.0
+Periodfill Mechanism	90.48 ± 0.56	92.41 ± 0.49	96.21 ± 0.45	39 ± 5	92.5
+KCF Module	92.15 ± 0.51	93.28 ± 0.44	96.35 ± 0.38	24 ± 3	95.3
+Conflict Coverage	93.67 ± 0.47	94.12 ± 0.41	96.52 ± 0.35	18 ± 3	91.8
+ADA Module	95.21 ± 0.44	94.68 ± 0.39	96.75 ± 0.33	13 ± 2	98.7

**Table 5:** Evaluation methodology.

Evaluation of Indicators	Descriptions
mAP	Reflects the model’s overall performance in target localization and classification. It effectively measures the model’s detection ability at different confidence thresholds, especially suitable for multi-class target detection tasks.
Precision	Measures the proportion of actual positive samples among those predicted as positive by the model, reflecting the model’s ability to avoid false positives.
Recall	Reflects the model’s ability to avoid false negatives. A high recall indicates the model’s capability to detect most targets, suitable for scenarios sensitive to missed detections.
MOTA	Considers the impact of false positives (FPs), false negatives (FNs), and identity switches (IDSWs) on locking performance. It provides a comprehensive reflection of the locking algorithm’s performance in real-world applications.
MOTP	Measures the positional accuracy of the locking boxes relative to the ground truth boxes, reflecting the accuracy of the target localization. A high MOTP value indicates a high overlap between the locking box and the true box.
IDF1	Measures the accuracy of identity retention, calculated based on precision and recall of identity matching.
IDSW	Tracks the number of identity switches during the locking process. Fewer IDSWs indicate better performance in maintaining target identity during locking.
FPS	Frames Per Second, measures the number of frames the algorithm processes per second, a key metric for evaluating the real-time performance of the algorithm.
AvgFrame	Represents the average number of frames during which the algorithm locks a target in operation.
MaxFrame	Represents the maximum number of frames during which the algorithm locks a target in operation.
AvgLockDur	Represents the average continuous locking time of the algorithm during operation.
AvgConsecutiveFrame	Measures the average number of frames the target is continuously locked, reflecting the algorithm’s ability to maintain stable continuous locking.
System Stability	Evaluates the locking system’s performance over extended periods of operation. High stability indicates the system can run continuously and reliably in complex environments.
Positioning Accuracy	Evaluates the locking system’s precision in responding to target positions, typically measured by servo angle errors.
Initialization Time	Evaluates the time taken for the locking system to transition from startup to a stable locking state. Low startup time indicates the system can quickly enter operational status, suitable for scenarios requiring high response speed.

## Data Availability

The data that support the findings of this study are available on request from the corresponding author. The data are not publicly available due to them containing information that could compromise research participant privacy.
